# Sedentary Behavior and Happiness: The Mediation Effects of Social Capital

**DOI:** 10.1093/geroni/igab044

**Published:** 2021-10-05

**Authors:** Akitomo Yasunaga, Mohammad Javad Koohsari, Ai Shibata, Kaori Ishii, Rina Miyawaki, Kuniko Araki, Koichiro Oka

**Affiliations:** 1 Faculty of Liberal Arts and Sciences, Bunka Gakuen University, Shibuya, Tokyo, Japan; 2 Faculty of Sport Sciences, Waseda University, Tokorozawa, Saitama, Japan; 3 Melbourne School of Population and Global Health, The University of Melbourne, Melbourne, Victoria, Australia; 4 Faculty of Health and Sport Sciences, University of Tsukuba, Tsukuba, Ibaraki, Japan; 5 School of Arts and Letters, Meiji University, Suginami, Tokyo, Japan

**Keywords:** Emotional well-being, Health promotion, Mental health, Sitting time, Social sustainability

## Abstract

**Background and Objectives:**

This study aimed to examine the associations between time spent in 6 different domains of sedentary behavior and happiness and whether social capital mediated such associations among adults and older adults living in a rural area of Japan.

**Research Design and Methods:**

Cross-sectional data from 3,357 participants (mean age: 60 ± 16 years) were used. 6 domains of sedentary behavior, happiness, and social capital were assessed using a self-report questionnaire. Age-stratified multivariable linear regression models adjusted for covariates were used to examine the associations between 6 domains of sedentary behavior and happiness. For relationships where the direct effect was significant, we tested the mediating effects of 2 social capital measures.

**Results:**

Among both adults and older adults, more time spent viewing television was significantly associated with lower happiness scores, and more time spent engaging in other leisure activities was significantly associated with higher happiness scores. In addition, more time spent using cell phones and computers was significantly associated with lower happiness scores among the adults. Engaging in activities with neighbors significantly mediated the relationship between other leisure activities and happiness in the adults and older adults, and between television viewing and happiness in the older adults.

**Discussion and Implications:**

Our findings indicated that less television viewing and more mentally active sedentary behavior (e.g., talking with others and engaging in hobbies) were associated with greater happiness. One aspect of social capital, engaging in activities with neighbors, acts as a potential mediator for relationships between sedentary behavior and happiness.

**Translational Significance:** Less engagement in passive and more engagement in mentally active sedentary behaviors were associated with greater happiness, which was fostered by participating in activities with neighbors. Reducing passive sedentary behavior, such as television viewing, could promote positive mental health in adults and older adults.

## Background and Objectives

Positive mental health, such as happiness, life satisfaction, optimism, and self-esteem, confers several health benefits. For example, a meta-analysis of longitudinal studies in the general population found that greater happiness was associated with decreased mortality risk (Martín-María et al., 2017). Another review suggested that positive mental health consistently protected against cardiovascular diseases, independent of traditional risk factors and ill-being (Boehm & Kubzansky, 2012). Assessments of positive mental health do not necessarily detect negative mental health status, such as anxiety, depression, and psychological distress, and there is discontinuity between positive mental health and mental illness (Diener & Emmons, 1984). However, previous mental health surveys have often solely focused on negative mental health (Winzer et al., 2014).

Sedentary behavior is defined as “any waking behavior characterized by an energy expenditure ≤1.5 metabolic equivalents, while in a sitting, reclining or lying posture” (Sedentary Behavior Research Network, 2012; Tremblay et al., 2017) and is one of the key behaviors influencing mental health. Previous studies have demonstrated that sedentary behavior has a detrimental effect on mental health (Huang et al., 2020; Yasunaga et al., 2018). For instance, a meta-analysis of observational studies found that longer time spent in sedentary behavior was associated with an increased risk of depression (Zhai et al., 2015). In a recent study conducted in the United States, older adults aged 65 years and older reported that television viewing, the most common sedentary leisure activity in later life, was associated with increased loneliness (Fingerman et al., 2021). Most previous studies examining associations between sedentary behavior and mental health evaluated negative aspects of participants’ mental health. Only a few studies have examined whether sedentary behavior is associated with positive aspects of mental health, such as happiness (Felez-Nobrega et al., 2021; Pengpid & Peltzer, 2019).

Notably, recent studies have reported that different types of sedentary behavior have distinct effects on mental health (Hallgren et al., 2018, 2020; Kikuchi et al., 2014). For example, a study conducted in Japan found that a lower amount of passive sedentary behavior (e.g., television viewing) and a greater amount of mentally active behavior (e.g., computer use, reading books, and desk-based office work) were associated with a decreased risk of depression in older adults (Kikuchi et al., 2014). Similar results were also reported by several studies conducted in Western countries (Hallgren et al., 2018, 2020). Therefore, it is important to consider not only the total sedentary time but also the impact of different types of sedentary behavior on mental health. However, it is not yet clear how the different sedentary behavior types are associated with positive mental health among a sample of adults and older people. In addition, researchers have suggested that patterns of sedentary behaviors are different between adults and older adults. For example, epidemiologic studies have shown that older adults spend more time watching television than adults (e.g., Sumimoto et al., 2021). Many older adults do not have full-time jobs because the retirement age in many Japanese companies is 60–65 years. Therefore, they are likely to spend less time sitting at work and more time engaging in leisure-related sedentary activities in their free time. Because older adults are less likely to own smartphones and computers, they might spend less time engaged in sedentary activities related to these devices. Thus, differences in patterns of sedentary behavior between adults and older adults are expected to have differential impacts on social capital and mental health (Fingerman et al., 2020). Furthermore, although researchers have suggested that people living in urban and rural areas have different health-related behavior patterns and health risks (e.g., Teckle et al., 2012), many studies examining the impact of sedentary behavior on health, including mental health, have been conducted for people living in and around urban areas. In 2019, it was estimated that 44% of the world’s population lives in rural areas (The World Bank, 2018). We need to accumulate evidence to plan strategies that promote health for people living in rural areas.

The social pathway through which sedentary behavior influences mental health is yet to be known. Social capital is one of the hypothesized links between sedentary behavior and positive mental health. Social capital is an ambiguous concept, but the existing literature highlights social cohesion and network theory as two distinct social capital conceptions (Kawachi, 2006). The social cohesion approaches emphasize the cognitive or structural side of social capital through questions about trust in others, perceptions of social belonging and integration, and civic or social participants’ levels. In contrast, network theory approaches rely on formal social network analysis methods to measure social resources and networks (Moore & Kawachi, 2017). Mounting evidence using both approaches has shown the health benefits of social capital (Ehsan et al., 2019; Murayama et al., 2012; Rodgers et al., 2019). Previous studies have found that higher social capital was positively associated with happiness, a representative indicator of positive mental health (Doherty & Kelly, 2010; Tsuruta et al., 2019). Although there have been studies showing that social capital influences sedentary behavior (e.g., Anderson et al., 2016), a few researchers have suggested reverse causation, where fostering social capital may also be affected by sedentary behavior through its influence on social interactions. Furthermore, different types of sedentary behavior may differentially affect these associations. For example, people who spend much time at home watching television or using their phones or computers will have less interaction with their neighbors, which will have a negative impact on social capital. A study reported that time spent walking dogs, but not sedentary behavior, was positively associated with the frequency of social interactions among mid-older people (Curl et al., 2020). On the other hand, people who spend more time in a sitting position related to driving or public transportation may go out more often, which may positively impact social capital. A study in Japan found that older drivers engaged in more physical activity than older nondrivers (Amagasa et al., 2018). The difference in the associations between various activities and social relationships might have an impact on mental health. Nevertheless, to our knowledge, no study has tested the mediating effects of social capital on the associations between various types of sedentary behavior and positive mental health.

This study aimed to examine the associations between time spent in each of six different sedentary behaviors and happiness among adults and older adults living in rural areas of Japan and whether social capital mediated such associations. The hypotheses were as follows: (a) a lower amount of passive sedentary behavior and a greater amount of mentally active sedentary behavior are associated with greater happiness, (b) various types of sedentary behavior between adults and older adults will have different effects on social capital and happiness, and (c) social capital is a possible mediator linking sedentary behavior and happiness.

## Research Design and Methods

### Participants and Procedures

This study was conducted in Minami-Izu town, Shizuoka Prefecture, Japan, as part of an extensive epidemiological study of this community. Minami-Izu town is a small country town located approximately 138 km southwest of Tokyo. In 2020, the town’s total population was approximately 8,100 residents, 48% of whom were older than 65 years. The population aged 65 years is nearly 20% higher than the average rate for Japan as a whole (approximately 29% in 2020). The rate of workers engaged in the primary sector of industry (agriculture, forestry and fishing) in Minami-Izu town is approximately 9.5% (Minami-Izu Town, 2020), which is approximately three times the average rate for Japan as a whole (approximately 3% in 2019; The Japan Institute for Labour Policy and Training, 2020). Data were collected between October and November 2016 from all adults older than 20 years living in Minami-Izu town. Research staff distributed the questionnaire to all adults (*n* = 7,360), except those hospitalized, bedridden, or institutionalized. The questionnaires were collected a couple of weeks later. Of the potential respondents, 4,714 residents returned the questionnaire. After excluding participants with missing data in the self-reported measures, including six domains of sedentary behavior, two social capital measures, happiness, and covariates, 3,357 participants were included in this study. All participants provided written informed consent. The Waseda University Research Ethics Committee (Japan) approved the study (2016-280).

### Measures

#### Social capital

Social capital was assessed in two dimensions, social cohesion and activities with neighbors, which corresponds to the network theory, using items in Mujahid and colleagues (2007). Social cohesion was measured using the following four items: “People around here are willing to help their neighbors,” “People in my neighborhood generally get along with each other,” “People in my neighborhood can be trusted,” and “People in my neighborhood share the same values.” The participants responded to these items on a Likert-type scale ranging from 1 (strongly agree) to 5 (strongly disagree). The measure activities with neighbors were measured using the following five items: “About how often do you and people in your neighborhood do favors for each other? By favors, we mean such things as watching each other’s children, helping with shopping, lending garden or house tools, and other small acts of kindness,” “When a neighbor is not at home or on vacation, how often do you and other neighbors watch over their property?,” “How often do you and other people in the neighborhood ask each other for advice about personal things such as child-rearing or job openings?,” “How often do you and people in your neighborhood have parties or other get togethers where other people in the neighborhood are invited?,” and “How often do you and other people in your neighborhood visit in each other’s homes or speak with each other on the street?” The participants responded to these items on a Likert-type scale ranging from 1 (often) to 4 (not at all). The response categories were reverse-coded and summed such that higher scores indicated higher social cohesion and activities with neighbors. The internal consistencies for the two variables were relatively high in both age groups (all Cronbach’s α were >.80).

#### Self-reported sedentary behavior in six different domains

Participants’ sedentary behavior was assessed using a self-report questionnaire developed by Ishii and colleagues (2018), which estimated the amount of time spent in each of the six sedentary behavior domains. The participants responded to daily average time spent in sedentary behavior in hours and minutes over the past 7 days for the following six domains: riding in a car as driver or passenger (hereafter referred to as “car”); using public transport (hereafter referred to as “public transport”); being at work (hereafter referred to as “work”); watching television, videos, and digital video discs (hereafter referred to as “television watching”); using a cell phone, tablet, or personal computer for nonwork purposes (hereafter referred to as “cell and computer use”); and sitting for other purposes in leisure time (e.g., talking, reading, listening to music, or engaging in a hobby) (hereafter referred to as “other leisure activities”). They were asked to provide a different response for workdays (or weekdays for unemployed individuals) and nonwork days (weekends). Mean workday and nonwork day values of total sedentary time were calculated by separately summing all six domains for workdays and nonwork days. Mean daily values of total sedentary time and each domain’s sedentary time were also calculated by a weighting of the number of workdays and nonwork days.

#### Happiness

The feeling of happiness was assessed using a single-item self-rated question, “How happy do you think of yourself at present?” The participants responded on a Likert-type scale ranging from 1 (unhappy), 2 (somewhat unhappy), 3 (somewhat happy), and 4 (happy).

#### Covariates

The following individual variables were considered potential confounders: age, gender (female or male), education (tertiary or higher or below tertiary), marital status (single or married), length of residence in the current address, and total sedentary behavior time.

### Statistical Analysis

**Figure 1.**
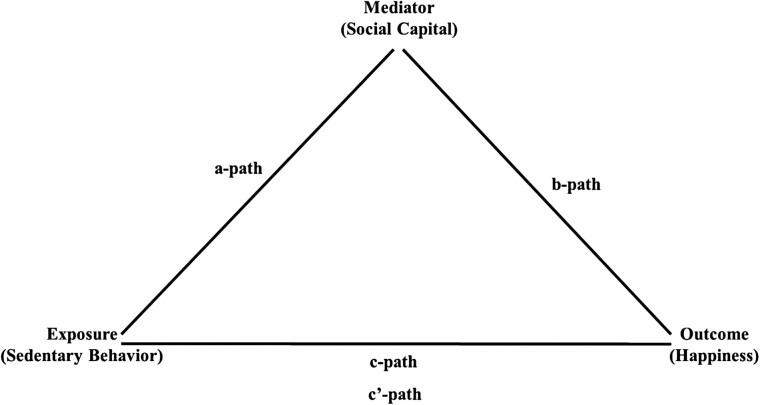
Framework for analyzing the relationships among six domains of sedentary behavior, social capital, and happiness.*Notes*: a-path = associations between time spent in six domains of sedentary behavior and two social capital measures; b-path = associations between two social capital measures and happiness; c-path = associations between time spent in six domains of sedentary behavior and happiness; c′-path = associations between time spent in six domains of sedentary behavior and happiness adjusting for social capital measures.

Descriptive analyses were used to calculate means and standard deviations or numbers and rates (%) for all variables in two age groups: adults who were 20–64 and older adults who were 65 years and older. Nonpaired *t* tests or chi-squared tests were conducted to examine the age differences of all variables. Figure 1 shows a framework for analyzing the associations between the six domains of sedentary behavior, social capital, and happiness. Age-stratified multivariable linear regression models were used to examine the associations of time spent in the six domains of sedentary behavior with happiness (direct effects; c-path). For associations where the direct effect was significant, we tested the mediating effects of two social capital measures in these relationships using a product-of-coefficients test (MacKinnon et al., 2002). Specifically, we calculated unstandardized partial regression coefficients (*B*) and 95% confidence intervals (95% CIs) in the following associations: (a) associations between time spent in the six domains of sedentary behavior and two social capital measures (a-path); (b) associations between two social capital measures (the potential mediators) and happiness (b-path); and (c) associations between time spent in the six domains of sedentary behavior and happiness adjusting for the social capital measures (c′-path). The indirect effects of the six domains of sedentary behavior and happiness through the potential mediators can be calculated as a product of the unstandardized coefficient (*B*) for the a-path and b-path (a × b). Hayes PROCESS was applied to test whether the indirect effect was significant (Hayes, 2013). The proportion of the effect that was mediated was calculated as [a × b/ (a × b + c′)] and is presented as percentages. All regression models were adjusted for covariates, and the six domains of sedentary behavior and two social capital measures were included in a separate model. All analyses were conducted using IBM SPSS Statistics 25.0 for Windows (IBM Japan Corp., Tokyo, Japan), and the level of significance was set at *p* < .05.

## Results

### Characteristics of Study Participants

Table 1 shows the characteristics of the participants. The mean age was 60.0 years, less than half were older adults aged 65 years and older, and more than half were female. There were significant differences between the adults and older adults in age, education, marital status, length of residence, social capital scores, time spent in five domains of sedentary behavior (car, work, television viewing, cell and computer use, and other leisure activities), and total sedentary time (*p* < .05).

**Table 1.** Characteristics of the Participants

Variable	20–64 years (*n* = 1,802)	65 years and older (*n* = 1,555)
	*N* (%) or mean (*SD*)	*N* (%) or mean (*SD*)
Age**	48.1 (11.6)	73.8 (7.2)
Gender		
Female	936 (51.9%)	811 (52.2%)
Male	866 (48.1%)	744 (47.8%)
Education**		
Tertiary or higher	855 (47.4%)	317 (20.4%)
Below tertiary	947 (52.6%)	1,238 (79.6%)
Marital status**		
Single	511 (28.4%)	220 (14.1%)
Married	1,291 (71.6%)	1,335 (85.9%)
Length of residence*	24.3 (17.4)	43.3 (22.7)
Social capital		
Social cohesion (range 0–20)	14.3 (2.6)	14.3 (2.7)
Activities with neighbors (range 0–20)**	10.5 (3.2)	11.6 (3.1)
Sedentary behavior (hours)		
Car**	1.2 (1.1)	0.8 (0.9)
Public transport	0.0 (0.3)	0.0 (0.3)
Work**	1.9 (2.3)	0.5 (1.4)
Television viewing**	3.1 (2.0)	3.8 (2.6)
Cell phone and computer use**	1.2 (1.3)	0.4 (1.0)
Other leisure activities**	1.0 (1.0)	1.3 (1.4)
Total**	8.4 (3.6)	6.9 (3.5)
Happiness	3.2 (0.6)	3.2 (0.6)

*Note*: Statistically significant difference (*p < .05, **p < .01) based on Pearson’s chi-squared or *t* test.

### Associations of Different Domains of Sedentary Behavior With Happiness (c-Path)

Table 2 shows the associations between the six domains of sedentary behavior and happiness. Among the adults, after adjusting for covariates, longer time spent on television viewing and cell phone and computer use were significantly associated with lower happiness scores (*B* = –0.018; 95% CI: –0.035, –0.001; *p* < .05 and *B* = –0.029; 95% CI: –0.055, –0.002; *p* < .05, respectively), and longer time spent in other leisure activities was significantly associated with higher happiness scores (*B* = 0.059; 95% CI: 0.030; 0.088; *p* < .01). Among the older adults, after adjusting for covariates, longer time spent on television viewing was significantly associated with lower happiness scores (*B* = –0.016; 95% CI: –0.032, –0.001; *p* < .05), and longer time spent in other leisure activities was significantly associated with higher happiness scores (*B* = 0.037; 95% CI: 0.013, 0.061; *p* < .01).

**Table 2.** Associations Between Six Different Domains of Sedentary Behavior and Happiness

Variable	20–64 years *B* (95% CI)	65 years and older *B* (95% CI)
Car	−0.001 (−0.028, 0.026)	0.009 (−0.025, 0.042)
Public transport	0.059 (−0.055, 0.173)	0.005 (−0.079, 0.090)
Work	0.008 (−0.008, 0.023)	0.001 (−0.021, 0.023)
Television viewing	−0.018 (−0.035, −0.001)*	−0.016 (−0.032, −0.001)*
Cell phone and computer use	−0.029 (−0.055, −0.002)*	−0.006 (−0.037, 0.025)
Other leisure activities	0.059 (0.030, 0.088)**	0.037 (0.013, 0.061)**

*Notes*: *B* = unstandardized partial regression coefficient; 95% CI = 95% confidence interval. All models were adjusted for age, gender, education, marital status, and total sedentary behavior time. Only one sedentary behavior variable was included per model plus covariates.

*p < .05. **p < .01.

### Associations of Different Domains of Sedentary Behavior With Social Capital Scores (a-Path)

As shown in Table 3, longer time spent in other leisure activities was significantly associated with higher scores for activities with neighbors among both the adults (*B* = 0.229; 95% CI: 0.081, 0.378; *p* < .01) and older adults (*B* = 0.255; 95% CI: 0.132, 0.378; *p* < .01). Conversely, longer time spent on television viewing was significantly associated with lower scores for activities with neighbors among the older adults (*B* = –0.161; 95% CI: –0.239, –0.082; *p* < .01).

**Table 3.** Indirect Effects of Six Different Domains of Sedentary Behavior on Happiness Through Social Capital

Variable	c′-Path *B* (95% CI)	a-Path *B* (95% CI)	b-Path *B* (95% CI)	Indirect effect (a × b) *B* (95% CI)	Proportion mediation (%)
20–64 years					
Television viewing					
Social cohesion	−0.017 (−0.033, −0.001)*	−0.006 (−0.078, 0.065)	0.059 (0.048, 0.069)**	0.000 (−0.005, 0.004)	—
Activities with neighbors	−0.018 (−0.034, −0.001)*	0.004 (−0.083, 0.091)	0.030 (0.021, 0.039)**	0.000 (−0.003, 0.003)	—
Cell phone and computer use					
Social cohesion	−0.032 (−0.057, −0.007)*	0.025 (−0.085, 0.136)	0.059 (0.048, 0.069)**	0.002 (−0.005, 0.008)	—
Activities with neighbors	−0.030 (−0.055, −0.004)*	−0.037 (−0.171, 0.098)	0.030 (0.021, 0.039)**	−0.001 (−0.005, 0.003)	—
Other leisure activities					
Social cohesion	0.054 (0.026, 0.082)**	0.066 (−0.056, 0.188)	0.059 (0.048, 0.069)**	0.004 (−0.004, 0.014)	—
Activities with neighbors	0.051 (0.023, 0.080)**	0.229 (0.081, 0.378)**	0.030 (0.021, 0.039)**	0.007 (0.002, 0.013)*	11.9
65 years and older					
Television viewing					
Social cohesion	−0.016 (−0.032, −0.002)*	0.004 (−0.066, 0.074)	0.045 (0.035, 0.056)**	0.000 (−0.003, 0.003)	—
Activities with neighbors	−0.011 (−0.027, 0.004)	−0.161 (−0.239, −0.082)**	0.031 (0.021, 0.040)**	−0.005 (−0.008, −0.002)*	31.9
Other leisure activities					
Social cohesion	0.039 (0.015, 0.062)**	−0.025 (−0.134, 0.085)	0.045 (0.035, 0.056)**	−0.001 (−0.008, 0.006)	—
Activities with neighbors	0.030 (0.006, 0.053)*	0.255 (0.132, 0.378)**	0.031 (0.021, 0.040)**	0.008 (0.003, 0.014)*	21.4

*Notes*: *B* = unstandardized partial regression coefficient; 95% CI = 95% confidence interval. c′-path = associations of time spent in six domains of sedentary behavior with happiness adjusted for covariates and potential mediators; a-path = associations time spent in six domains of sedentary behavior with potential mediators adjusted for covariates; b-path = associations of potential mediators with happiness adjusted for covariates. Covariates were age, gender, education, marital status, length of residence in the current address, and total sedentary behavior time.

*p < .05. **p < .01.

### Associations of Social Capital Scores With Happiness (b-Path)

As shown in Table 3, higher social cohesion scores were significantly associated with higher happiness scores among both the adults (*B* = 0.059; 95% CI: 0.048, 0.069; *p* < .01) and older adults (*B* = 0.045; 95% CI: 0.035, 0.056; *p* < .01). Similarly, higher scores for activities with neighbors were significantly associated with higher happiness scores among the adults (*B* = 0.030; 95% CI: 0.021, 0.039; *p* < .01) and older adults (*B* = 0.031; 95% CI: 0.021, 0.040; *p* < .01).

### Mediated Pathways

Estimated mediation effects are shown in Table 3. The indirect effects of time spent in other leisure activities on happiness scores through the activities with neighbors scores were significant among both the adults (*B* = 0.007; 95% CI: 0.002, 0.013; *p* < .05) and older adults (*B* = 0.008; 95% CI: 0.003, 0.014; *p* < .05). The proportions of the total effect of time spent in other leisure activities on happiness scores among the adults and older adults mediated by the activities with neighbors scores were 11.9% and 21.4%, respectively. The indirect effect of time spent on television viewing on happiness scores through the activities with neighbors scores was significant among the older adults (*B* = –0.005; 95% CI: –0.008, –0.002; *p* < .05). The activities with neighbors score mediated 31.9% of the relationship.

## Discussion

This was the first study, to our knowledge, to examine how different types of sedentary behavior were associated with happiness among Asian adults and older adults and to test the meditating effects of social capital in these relationships. We confirmed the hypotheses established based on previous studies: (a) a lower amount of passive sedentary behavior and a greater amount of mentally active sedentary behavior were associated with greater happiness, (b) various types of sedentary behavior between the adults and older adults had differential effects on social capital and happiness, and (c) social capital was a possible mediator between sedentary behavior and happiness.

As expected, our data showed that the older adults spent more time engaging in television viewing and leisure-related sedentary activities than the adults. The adults spent more time engaged in sedentary behavior related to work, cars, and cell phone and computer use than the older adults. Additionally, the characteristics of the sedentary behavior of our participants living in a rural area of Japan were as follows when compared with other studies conducted in Japan, which used the same questionnaire as this study to assess the amount of time spent engaged in each of six different sedentary behaviors. Compared to the median sedentary behavior time among adults aged 40–64 years living in urban and rural core cities reported by Ishii and colleagues (2018), the median sedentary behavior times among our adult participants were higher for the car (1.0 hr/day vs 0.1 hr/day) and television viewing (2.9 hr/day vs 1.8 hr/day) measures and lower for the public transportation (almost 0 hr/day vs 0.2 hr/day) and work (0.7 hr/day vs 1.3 hr/day) measures. The median times for the cell phone and computer use (0.9 hr/day vs 1.1 hr/day) and other leisure activities (0.8 hr/day vs 1.0 hr/day) measures were almost the same between the two samples. Compared with the mean sedentary behavior time for older adults aged 65–84 years living in urban areas reported by Shibata and colleagues (2019), the mean sedentary behavior times for our older participants were higher for the television viewing measure (3.8 hr/day vs 3.3 hr/day) and lower for the public transportation (almost 0 hr/day vs 0.3 hr/day), cell phone and computer use (0.4 hr/day vs 0.7 hr/day), and other leisure activities (1.3 hr/day vs 1.8 hr/day) measures. The mean times for the car (0.8 hr/day vs 0.7 hr/day) and work (0.5 hr/day vs 0.4 hr/day) measures were almost identical between the two samples. As a super-aged society, the population is rapidly declining in Japan, especially in rural areas. Therefore, public transportation is inadequate, and there are few entertainment facilities in the neighborhood (Koohsari et al., 2018). Minami-Izu town, where this survey was conducted, is no exception. There are few public transportation systems, such as buses and trains, and almost no shopping centers or amusement facilities. These poor urban design features may lead to longer times spent in sedentary behavior that are related to cars and television viewing. On the other hand, similar to other rural towns in Japan, many people are engaged in the primary industries in Minami-Izu town, which would be associated with lower work-related sedentary behavior time in the adults. Future research needs to statistically examine the interactions between patterns of sedentary behavior and different sizes of cities using extracted representative samples from regions of different sizes, which included rural and urban areas.

Kikuchi and colleagues (2014) categorized sedentary behavior into passive (e.g., television viewing) and mentally active (e.g., computer use, reading books, and desk-based office work) behaviors. They examined the impact of each on health. Recent studies have reported different effects of the two different types of sedentary behavior on negative mental health: less time spent in passive sedentary behavior and more time spent in mentally active sedentary behavior were associated with lower depression (Hallgren et al., 2018; Huang et al., 2020). Consistent with these studies, we found that more time spent on television viewing, a representative passive sedentary behavior, was associated with worse happiness among adults and older adults. Our findings suggested that reducing television viewing time, which is the largest portion of total time spent in sedentary behavior, might improve happiness, a positive psychological indicator, and negative mental health. Our results also showed that more time spent engaged in cell and computer use was associated with less happiness among the adults. In a study by Kikuchi and colleagues (2014), computer use was considered mentally active behavior that was hypothesized to positively affect mental health. Previous findings of associations between computer use and mental health are mixed. Some previous reviews and meta-analyses have suggested that computer use could increase the risk of depression (Teychenne et al., 2010; Zhai et al., 2015). One possible reason for the mixed results could be the influence of using computers and cell phones. Our study asked participants to report daily average time spent using a computer, cell phone, or tablet computer for nonwork purposes. Thus, almost all computer use may have included passive sedentary behavior, such as web surfing, that does not require cognitive effort. These measures may have shown a negative association with mental health. Further studies are needed to ask in detail the purpose of computer use (e.g., watching videos, web surfing, or research). In addition, there was no association between cell phone and computer use and happiness among the older adults. This is probably due to the low ownership rate of cell phones and computers and the short time spent using them among the older adults. Similar to previous findings (Hallgren et al., 2018; Huang et al., 2020), we found that time spent in leisure time sedentary behavior (e.g., talking with others, reading books, listening to music, and engaging in a hobby) was positively associated with happiness among both the adults and older adults. Our findings suggested that engaging in activities that involve intellectual curiosity and social interactions, even sedentary behavior, may lead to greater happiness.

There are several plausible explanations for the observed associations between sedentary behavior and positive mental health. Mentally active sedentary behavior requiring cognitive effort, such as reading and researching, may improve cognitive abilities (Sörman et al., 2018), and better cognitive abilities may lead to better positive mental health (Tan et al., 2019). Additionally, because active people have been reported to have higher positive mental health (Piqueras et al., 2011; Richards et al., 2015), reducing harmful sedentary behavior and increasing physical activity would enhance positive mental health. This study assumed that social capital would be a possible principal mediator linking sedentary behavior and happiness. We found that engaging in activities with neighbors, which is one aspect of social capital, mediated the observed associations between television viewing and happiness among the older adults and between other sedentary leisure activities with happiness among both the adults and older adults. Our findings showed that longer time spent viewing television was related to lower engagement in activities with neighbors, which in turn led to lower happiness. There are trade-offs between what activities people spend their time on. Therefore, if people spend a long time watching television, they will naturally spend less time in activities involving social contact. Fewer social relationships and activities have been shown to lead to lower happiness (Hsu et al., 2016; Tsuruta et al., 2019). This association was found only among the older adults because television viewing is a typical sedentary behavior in late life (Fingerman et al., 2021), and older adults spend more time viewing television than adults. Conversely, our findings showed that longer time spent engaged in leisure-related sedentary activities was related to a greater amount of interaction with others, leading to higher happiness. Talking to others and engaging in hobbies will likely increase social contact in both the real world and on the internet. Again, social contact has been found to have a positive impact on happiness (Hsu et al., 2016; Tsuruta et al., 2019). Our findings suggested that reducing the time spent viewing television and increasing the time spent engaged in social and intellectual activities may lead to greater happiness by fostering engagement with neighbors and their activities, which is one aspect of social capital.

However, social cohesion was not found to be a mediator in the association between sedentary behavior and happiness in the adults and older adults. Our results showed that social cohesion impacted happiness among the adults and older adults, but there were no dimensions of sedentary behavior that were associated with social cohesion. Network theory focuses on the individual attribute of social capital, whereas social cohesion emphasizes the collective attributes of social capital (Kawachi, 2006). Although this point needs to be further evaluated, sedentary behavior may not foster the type of social cohesion that represents communitarian concept of society.

This study has some limitations. First, we cannot infer a cause-and-effect relationship between different types of sedentary behavior and happiness as this was a cross-sectional study. Second, self-reported sedentary time may not accurately reflect actual sedentary time due to recall and social desirability biases. Although further research is necessary to use more rigorous methods, our findings provide initial evidence to inform future studies with regard to the relationships among different sedentary behaviors, positive mental health, and social capital.

### Implications

This study provides evidence of the associations between different types of sedentary behavior and happiness: less time engaging in passive sedentary behavior, for example, television viewing, and more time engaging in mentally active sedentary behavior, such as talking with others and engaging in a hobby, are associated with greater happiness. In addition, one aspect of social capital, involvement in activities with neighbors, might act as a potential mediator for some relationships between sedentary behavior and happiness. These findings may help promote positive mental health.

## Data Availability

The data that support the findings of this study are available from the corresponding author upon reasonable request. The study was not preregistered.
